# ggVennDiagram: Intuitive Venn diagram software extended

**DOI:** 10.1002/imt2.177

**Published:** 2024-02-14

**Authors:** Chun‐Hui Gao, Chengjie Chen, Turgut Akyol, Adrian Dusa, Guangchuang Yu, Bin Cao, Peng Cai

**Affiliations:** ^1^ National Key Laboratory of Agricultural Microbiology Huazhong Agricultural University Wuhan China; ^2^ College of Resources and Environment Huazhong Agricultural University Wuhan China; ^3^ School of Civil and Environmental Engineering and Singapore Centre for Environmental Life Sciences Engineering Nanyang Technological University Singapore Singapore; ^4^ State Key Laboratory for Conservation and Utilization of Subtropical Agro‐Bioresources, College of Horticulture South China Agricultural University Guangzhou China; ^5^ Department of Molecular Biology and Genetics Aarhus University Aarhus Denmark; ^6^ Department of Sociology University of Bucharest Bucharest Romania; ^7^ Department of Bioinformatics, School of Basic Medical Sciences Southern Medical University Guangzhou China; ^8^ Hubei Key Laboratory of Soil Environment and Pollution Remediation Huazhong Agricultural University Wuhan China

## Abstract

Highlights of ggVennDiagram include: (1) Subset/Region filling Venn diagram up to seven sets; (2) Upset plot with unlimited sets; (3) Venn Calculator for two or more sets; (4) Provide as R package, Shiny App, and TBtools plugin.

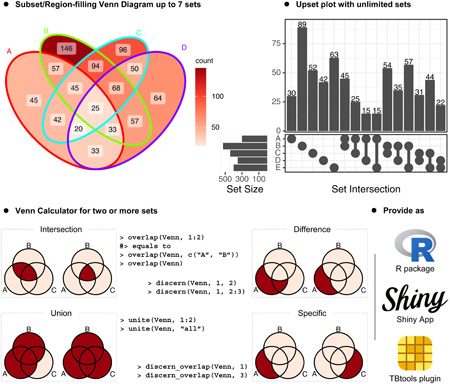

To the Editor,

Venn diagrams are widely used to illustrate relationships among multiple datasets. As one of the most popular data visualization platforms, the R programming language stands out with numerous packages, including VennDiagram, venn, and RVenn, designed for plotting Venn diagrams [1, 2, 3]. Building upon the foundations laid by these existing tools, we developed ggVennDiagram [4]. Due to the support for the grammar of graphics, precise region/subset filling, and some other easy‐to‐use features, the ggVennDiagram package has emerged as one of the most favored tools in this domain.

Over the past 2 years, ggVennDiagram has undergone continuous refinement, benefiting from a number of functional optimizations, and earned more than 100 literature citations. In this study, we release a milestone version 1.5 that includes the following new features.

## EASIER FOR NEW INSTALLATION THAN BEFORE

Before version 1.5, ggVennDiagram had a large package dependency tree, which contains more than 90 packages. Among them, the sf package emerged as the heaviest package. It not only has the largest package size but also has several system requirements, which are often not installed by most users. It was a primary cause for installation failures, as highlighted in GitHub Issues (https://github.com/gaospecial/ggVennDiagram/issues). However, sf is essential for shape generation in ggVennDiagram. Removing it directly is not feasible. Taking this into consideration, we relocated the shape generation functions to a new package, namely shapeMageR. Furthermore, several other dependencies, including RVenn, purrr, magrittr, and plotly were also removed (see Table S1). Consequently, the total file size of ggVennDiagram, together with its package dependencies, has been substantially reduced from 210 MB (1.1.0) to 36 MB in the current version, as been analyzed by the pak package manager [5] (see File S1). This makes the installation of ggVennDiagram much easier than before.

## NATIVE SUPPORT OF UPSET PLOT

**Figure 1:**
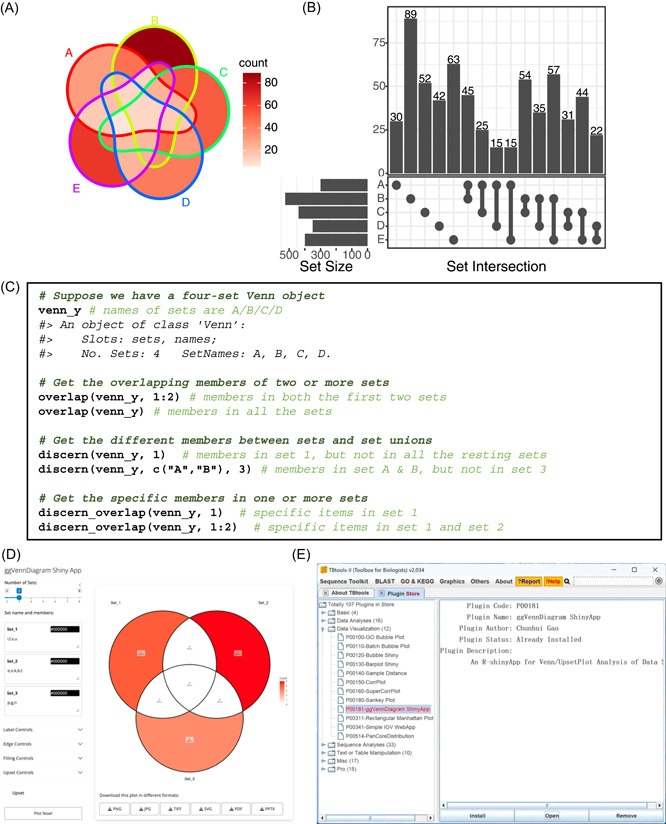
Features of ggVennDiagram. Venn diagram (A) and upset plot (B) with five sets input. (A) The numbers of subset members can be distinguished by light and deep red color fillings. (B) Top plot shows the size of intersections; left plot shows the size of five sets; main plot shows what these intersections come from. Intersections and sets were sorted alphabetically. (C) Example of Venn calculator. User can easily access the subsets with Venn object and generic methods. A reproducible example is provided in File S1. Screenshot of the ggVennDiagram Shiny App (D) and ggVennDiagram plugin in TBtools (E).

Venn Diagrams and Upset plot are two types of visualizing methods that are generally used under the same scenario. Plotting Upset plot is supported by the UpsetR package in the R platform [6]. However, we believe it would be more convenient to combine Upset method together with the Venn Diagram. Therefore, we introduced native support for Upset plots in ggVennDiagram. The implementation of Upset plot is inspired by the aplot package [7], which enables automatic axis alignments between subplots. We first create subplots of Upset plot, and subsequently combine the three components together. Figure 1 shows the Venn diagram and Upset plot generated by ggVennDiagram. Please note that the shapes for this five set diagram, as well as those for six and seven sets, are imported from the original package “venn,” authored by Adrian Dușa [3].

## FULL FUNCTIONAL VENN CALCULATOR

The S4 Venn class were implemented to store structured sets data such as the set members and set names. A series of methods were implemented for enabling set calculation of the Venn object. As shown in Figure 1C, we can get the overlapping, different and specific members of one or more sets by using these methods. Thus, these functions provide a Venn Calculator for further in‐depth analysis of sets data.

## OFFICIAL SHINY APP AND TBTOOLS PLUGIN

Shiny is a web application framework that allows developer to create interactive web‐based data visualization tools. The ggVennDiagram Shiny app was developed and deployed to *shinyapps.io*, a cloud‐based service provided by Posit/RStudio. Most of the parameters required for plotting a Venn Diagram are provided on the sidebar. Specifically, the exported vector‐format figures (svg, pdf, and pptx) can be further edited in place with handy tools, such as Adobe Illustrator, Microsoft PowerPoint, and so on (Figure 1D). TBtools is a bioinformatics software developed by Dr. Chengjie Chen [8]. It serves as a comprehensive toolset for completing multiple popular bioinformatics tasks. The plugin store provided in TBtools‐II makes it possible to integrate the Shiny app (Figure 1E) into the platform [9]. Those new features produced an interactive user interface, allowing light users who have limited coding abilities to use and generate high‐quality plots with ggVennDiagram.

## AUTHOR CONTRIBUTIONS

Chun‐Hui Gao and Bin Cao wrote this paper. Chun‐Hui Gao implemented this package. Chengjie Chen implemented the TBtools plugin. Turgut Akyol provided a prototype code for set operations. Adrian Dusa is the author and copyright holder for the polygon coordinates for enabling 5–7 sets Venn diagrams. Guangchuang Yu provided a tool chain and suggestions in programming. Peng Cai supervised the project. All authors have read the final manuscript and approved it for publication.

## CONFLICT OF INTEREST STATEMENT

The authors declare no conflict of interest.

## Supporting information


**Data S1:** Analysis of ggVennDiagram dependency.
**Data S2:** Example for Venn Calculator.

**Table S1.** Direct dependencies of ggVennDiagram version 1.1.

## Data Availability

The data that support the findings of this study are openly available in GitHub at https://github.com/gaospecial/ggVennDiagram. The ggVennDiagram R package is open source and freely available on CRAN (https://cran.r-project.org/package=ggVennDiagram) and GitHub (https://github.com/gaospecial/ggVennDiagram). The ggVennDiagram Shiny app can be accessed at Shinyapps.io (https://bio-spring.shinyapps.io/ggVennDiagram). The TBtools plugin can be accessed through its plugin store. Supplementary materials (figures, tables, scripts, graphical abstract, slides, videos, Chinese translated version, and update materials) may be found in the online DOI or iMeta Science http://www.imeta.science/.
