# Supplementary material for: ggVennDiagram: Intuitive Venn diagram software extended
Source: Imeta. 2024 Feb 14;3(1):e177. doi: 10.1002/imt2.177 (PMC10989133; doi:10.1002/imt2.177)
Supplement: Supplementary file 1 — Data S1: Analysis of ggVennDiagram dependency. Data S2: Example for Venn Calculator. [file IMT2-3-e177-s002.docx]

Supplementary Materials for

**ggVennDiagram:** **intuitive Venn diagram software extended**

**Chun-Hui Gao^1,2,3^, Chengjie Chen^4^, Turgut Akyol^5^, Adrian Dusa^6^,
Guangchuang Yu^7^, Bin Cao^3^, Peng Cai^1,2,8*^**

^1^ National Key Laboratory of Agricultural Microbiology, Huazhong Agricultural University, Wuhan 430070, China

^2^ College of Resources and Environment, Huazhong Agricultural University, Wuhan 430070, China

^3^ School of Civil and Environmental Engineering and Singapore Centre for Environmental Life Sciences Engineering, Nanyang Technological University, Singapore 639798, Singapore

^4^ State Key Laboratory for Conservation and Utilization of Subtropical Agro-Bioresources, College of Horticulture, South China Agricultural University, Guangzhou 510640, China

^5^ Department of Molecular Biology and Genetics, Aarhus University, Aarhus 8000, Denmark

^6^ Department of Sociology, University of Bucharest, Bucharest 050663, Romania

^7^ Department of Bioinformatics, School of Basic Medical Sciences, Southern Medical University, Guangzhou 510515, China

^8^ Hubei Key Laboratory of Soil Environment and Pollution Remediation, Huazhong Agricultural University, Wuhan 430070, China

**Running title**: ggVennDiagram v1.5

*** Correspondence:** [cp@mail.hzau.edu.cn](mailto:cp@mail.hzau.edu.cn) (Peng Cai)

**Keywords: Venn plot, Upset plot, data visualization, R package, Shiny App, TBtools**

List of Supplementary Materials

[Data S1 Analysis of ggVennDiagram dependency 3](#_Toc156847554)

[Heavy dependency of prior version of ggVennDiagram 3](#_Toc156847555)

[Current dependency of ggVennDiagram 9](#_Toc156847556)

[History of dependencies of ggVennDiagram 10](#_Toc156847557)

[Data S2 Example for Venn Calculator 13](#_Toc156847558)

# Data S1 Analysis of ggVennDiagram dependency

## Heavy dependency of prior version of ggVennDiagram

ggVennDiagram has a large package dependencies. This is because we build this package standing on the shoulder of those who came before us. Although we only import ten packages in the development, the dependence tree is huge.

deps = pak**::pkg_deps_tree**("gaospecial/ggVennDiagram@V1.1")
*#> ℹ Loading metadata database✔ Loading metadata database ... done*
*#> gaospecial/ggVennDiagram@V1.1 1.1.0 ✨👷🏻‍♂️🔧*
*#> ├─sf 1.0-15 ✨ ⬇ (86.91 MB)*
*#> │ ├─classInt 0.4-10 ✨*
*#> │ │ ├─e1071 1.7-14 ✨ ⬇ (662.46 kB)*
*#> │ │ │ ├─class 7.3-22*
*#> │ │ │ │ └─MASS 7.3-60 -> 7.3-60.0.1 🚀 ⬇ (1.18 MB)*
*#> │ │ │ └─proxy 0.4-27 ✨*
*#> │ │ ├─class*
*#> │ │ └─KernSmooth 2.23-22*
*#> │ ├─DBI 1.2.1 ✨👷🏻‍♂️ ⬇ (1.12 MB)*
*#> │ ├─magrittr 2.0.3 ✨*
*#> │ ├─Rcpp 1.0.12 ✨ ⬇ (3.31 MB)*
*#> │ ├─s2 1.1.6 ✨ ⬇ (11.91 MB)*
*#> │ │ ├─Rcpp*
*#> │ │ └─wk 0.9.1 ✨ ⬇ (1.97 MB)*
*#> │ └─units 0.8-5 ✨ ⬇ (958.47 kB)*
*#> │ └─Rcpp*
*#> ├─ggplot2 3.4.4 ✨*
*#> │ ├─cli 3.6.2 ✨*
*#> │ ├─glue 1.7.0 ✨ ⬇ (159.26 kB)*
*#> │ ├─gtable 0.3.4 ✨*
*#> │ │ ├─cli*
*#> │ │ ├─glue*
*#> │ │ ├─lifecycle 1.0.4 ✨*
*#> │ │ │ ├─cli*
*#> │ │ │ ├─glue*
*#> │ │ │ └─rlang 1.1.3 ✨ ⬇ (1.89 MB)*
*#> │ │ └─rlang*
*#> │ ├─isoband 0.2.7 ✨*
*#> │ ├─lifecycle*
*#> │ ├─MASS*
*#> │ ├─mgcv 1.9-0 -> 1.9-1 🚀 ⬇ (3.65 MB)*
*#> │ │ ├─nlme 3.1-163 -> 3.1-164 🚀 ⬇ (2.42 MB)*
*#> │ │ │ └─lattice 0.21-9 -> 0.22-5 🚀 ⬇ (1.37 MB)*
*#> │ │ └─Matrix 1.6-4 -> 1.6-5 🚀 ⬇ (5.36 MB)*
*#> │ │ └─lattice*
*#> │ ├─rlang*
*#> │ ├─scales 1.3.0 ✨*
*#> │ │ ├─cli*
*#> │ │ ├─farver 2.1.1 ✨*
*#> │ │ ├─glue*
*#> │ │ ├─labeling 0.4.3 ✨*
*#> │ │ ├─lifecycle*
*#> │ │ ├─munsell 0.5.0 ✨*
*#> │ │ │ └─colorspace 2.1-0 ✨*
*#> │ │ ├─R6 2.5.1 ✨*
*#> │ │ ├─RColorBrewer 1.1-3 ✨*
*#> │ │ ├─rlang*
*#> │ │ └─viridisLite 0.4.2 ✨*
*#> │ ├─tibble 3.2.1 ✨*
*#> │ │ ├─fansi 1.0.6 ✨*
*#> │ │ ├─lifecycle*
*#> │ │ ├─magrittr*
*#> │ │ ├─pillar 1.9.0 ✨*
*#> │ │ │ ├─cli*
*#> │ │ │ ├─fansi*
*#> │ │ │ ├─glue*
*#> │ │ │ ├─lifecycle*
*#> │ │ │ ├─rlang*
*#> │ │ │ ├─utf8 1.2.4 ✨*
*#> │ │ │ └─vctrs 0.6.5 ✨*
*#> │ │ │ ├─cli*
*#> │ │ │ ├─glue*
*#> │ │ │ ├─lifecycle*
*#> │ │ │ └─rlang*
*#> │ │ ├─pkgconfig 2.0.3 ✨*
*#> │ │ ├─rlang*
*#> │ │ └─vctrs*
*#> │ ├─vctrs*
*#> │ └─withr 3.0.0 ✨ ⬇ (240.48 kB)*
*#> ├─dplyr 1.1.4 ✨*
*#> │ ├─cli*
*#> │ ├─generics 0.1.3 ✨*
*#> │ ├─glue*
*#> │ ├─lifecycle*
*#> │ ├─magrittr*
*#> │ ├─pillar*
*#> │ ├─R6*
*#> │ ├─rlang*
*#> │ ├─tibble*
*#> │ ├─tidyselect 1.2.0 ✨*
*#> │ │ ├─cli*
*#> │ │ ├─glue*
*#> │ │ ├─lifecycle*
*#> │ │ ├─rlang*
*#> │ │ ├─vctrs*
*#> │ │ └─withr*
*#> │ └─vctrs*
*#> ├─stringr 1.5.1 ✨*
*#> │ ├─cli*
*#> │ ├─glue*
*#> │ ├─lifecycle*
*#> │ ├─magrittr*
*#> │ ├─rlang*
*#> │ ├─stringi 1.8.3 ✨*
*#> │ └─vctrs*
*#> ├─magrittr*
*#> ├─purrr 1.0.2 ✨*
*#> │ ├─cli*
*#> │ ├─lifecycle*
*#> │ ├─magrittr*
*#> │ ├─rlang*
*#> │ └─vctrs*
*#> ├─tibble*
*#> ├─plotly 4.10.4 ✨ ⬇ (3.20 MB)*
*#> │ ├─ggplot2*
*#> │ ├─scales*
*#> │ ├─httr 1.4.7 ✨*
*#> │ │ ├─curl 5.2.0 ✨ ⬇ (813.18 kB)*
*#> │ │ ├─jsonlite 1.8.8 ✨*
*#> │ │ ├─mime 0.12 ✨*
*#> │ │ ├─openssl 2.1.1 ✨*
*#> │ │ │ └─askpass 1.2.0 ✨*
*#> │ │ │ └─sys 3.4.2 ✨*
*#> │ │ └─R6*
*#> │ ├─jsonlite*
*#> │ ├─magrittr*
*#> │ ├─digest 0.6.34 ✨ ⬇ (287.92 kB)*
*#> │ ├─viridisLite*
*#> │ ├─base64enc 0.1-3 ✨*
*#> │ ├─htmltools 0.5.7 ✨*
*#> │ │ ├─base64enc*
*#> │ │ ├─digest*
*#> │ │ ├─ellipsis 0.3.2 ✨*
*#> │ │ │ └─rlang*
*#> │ │ ├─fastmap 1.1.1 ✨*
*#> │ │ └─rlang*
*#> │ ├─htmlwidgets 1.6.4 ✨*
*#> │ │ ├─htmltools*
*#> │ │ ├─jsonlite*
*#> │ │ ├─knitr 1.45 ✨*
*#> │ │ │ ├─evaluate 0.23 ✨*
*#> │ │ │ ├─highr 0.10 ✨*
*#> │ │ │ │ └─xfun 0.41 ✨*
*#> │ │ │ ├─xfun*
*#> │ │ │ └─yaml 2.3.8 ✨*
*#> │ │ ├─rmarkdown 2.25 ✨*
*#> │ │ │ ├─bslib 0.6.1 ✨*
*#> │ │ │ │ ├─base64enc*
*#> │ │ │ │ ├─cachem 1.0.8 ✨*
*#> │ │ │ │ │ ├─rlang*
*#> │ │ │ │ │ └─fastmap*
*#> │ │ │ │ ├─htmltools*
*#> │ │ │ │ ├─jquerylib 0.1.4 ✨*
*#> │ │ │ │ │ └─htmltools*
*#> │ │ │ │ ├─jsonlite*
*#> │ │ │ │ ├─lifecycle*
*#> │ │ │ │ ├─memoise 2.0.1 ✨*
*#> │ │ │ │ │ ├─rlang*
*#> │ │ │ │ │ └─cachem*
*#> │ │ │ │ ├─mime*
*#> │ │ │ │ ├─rlang*
*#> │ │ │ │ └─sass 0.4.8 ✨*
*#> │ │ │ │ ├─fs 1.6.3 ✨*
*#> │ │ │ │ ├─rlang*
*#> │ │ │ │ ├─htmltools*
*#> │ │ │ │ ├─R6*
*#> │ │ │ │ └─rappdirs 0.3.3 ✨*
*#> │ │ │ ├─evaluate*
*#> │ │ │ ├─fontawesome 0.5.2 ✨*
*#> │ │ │ │ ├─rlang*
*#> │ │ │ │ └─htmltools*
*#> │ │ │ ├─htmltools*
*#> │ │ │ ├─jquerylib*
*#> │ │ │ ├─jsonlite*
*#> │ │ │ ├─knitr*
*#> │ │ │ ├─stringr*
*#> │ │ │ ├─tinytex 0.49 ✨*
*#> │ │ │ │ └─xfun*
*#> │ │ │ ├─xfun*
*#> │ │ │ └─yaml*
*#> │ │ └─yaml*
*#> │ ├─tidyr 1.3.0 ✨*
*#> │ │ ├─cli*
*#> │ │ ├─dplyr*
*#> │ │ ├─glue*
*#> │ │ ├─lifecycle*
*#> │ │ ├─magrittr*
*#> │ │ ├─purrr*
*#> │ │ ├─rlang*
*#> │ │ ├─stringr*
*#> │ │ ├─tibble*
*#> │ │ ├─tidyselect*
*#> │ │ └─vctrs*
*#> │ ├─RColorBrewer*
*#> │ ├─dplyr*
*#> │ ├─vctrs*
*#> │ ├─tibble*
*#> │ ├─lazyeval 0.2.2 ✨*
*#> │ ├─rlang*
*#> │ ├─crosstalk 1.2.1 ✨*
*#> │ │ ├─htmltools*
*#> │ │ ├─jsonlite*
*#> │ │ ├─lazyeval*
*#> │ │ └─R6*
*#> │ ├─purrr*
*#> │ ├─data.table 1.14.10 ✨ ⬇ (2.37 MB)*
*#> │ └─promises 1.2.1 ✨*
*#> │ ├─fastmap*
*#> │ ├─later 1.3.2 ✨*
*#> │ │ ├─Rcpp*
*#> │ │ └─rlang*
*#> │ ├─magrittr*
*#> │ ├─R6*
*#> │ ├─Rcpp*
*#> │ └─rlang*
*#> ├─RVenn 1.1.0 ✨*
*#> │ ├─ggforce 0.4.1 ✨*
*#> │ │ ├─ggplot2*
*#> │ │ ├─Rcpp*
*#> │ │ ├─scales*
*#> │ │ ├─MASS*
*#> │ │ ├─tweenr 2.0.2 ✨*
*#> │ │ │ ├─farver*
*#> │ │ │ ├─magrittr*
*#> │ │ │ ├─rlang*
*#> │ │ │ └─vctrs*
*#> │ │ ├─gtable*
*#> │ │ ├─rlang*
*#> │ │ ├─polyclip 1.10-6 ✨*
*#> │ │ ├─tidyselect*
*#> │ │ ├─withr*
*#> │ │ ├─lifecycle*
*#> │ │ ├─cli*
*#> │ │ ├─vctrs*
*#> │ │ └─systemfonts 1.0.5 ✨*
*#> │ ├─ggplot2*
*#> │ ├─magrittr*
*#> │ ├─purrr*
*#> │ ├─rlang*
*#> │ ├─vegan 2.6-4 ✨*
*#> │ │ ├─permute 0.9-7 ✨*
*#> │ │ ├─lattice*
*#> │ │ ├─MASS*
*#> │ │ ├─cluster 2.1.4 -> 2.1.6 🚀 ⬇ (615.06 kB)*
*#> │ │ └─mgcv*
*#> │ └─pheatmap 1.0.12 ✨*
*#> │ ├─RColorBrewer*
*#> │ ├─scales*
*#> │ └─gtable*
*#> ├─tidyr*
*#> └─venn 1.12 ✨ ⬇ (309.18 kB)*
*#> └─admisc 0.34 ✨ ⬇ (366.37 kB)*
*#>*
*#> Key: ✨ new | 🚀 update | ⬇ download | 👷🏻‍♂️ build | 🔧 compile*

deps **|>** **select**(package, filesize) **|>**
 **arrange**(**desc**(filesize))
*#> # A data frame: 92 × 2*
*#> package filesize*
*#> <chr> <int>*
*#> 1 sf 86909904*
*#> 2 stringi 14767028*
*#> 3 s2 11910183*
*#> 4 systemfonts 6555807*
*#> 5 bslib 6241176*
*#> 6 Matrix 5355734*
*#> 7 ggplot2 4309115*
*#> 8 ggforce 4302218*
*#> 9 mgcv 3650233*
*#> 10 Rcpp 3314542*
*#> # ℹ 82 more rows*

Among them, sf is the heaviest package that ggVennDiagram depend on.

Package sf not only has the largest size, but also depends on several system packages, such as GDAL, GEOS, PROJ, and so on. Furthermore, two of the sf-dependent packages, s2 and units also have system requirements, especially units, whose system dependence udunits-2 is an additional one that usually not installed by most of the ggVennDiagram users.

deps_sf = pak**::pkg_deps**("sf")
deps **|>** **filter**(package **%in%** deps_sf**$**package, sysreqs **!=** "") **|>**
 **select**(package, sysreqs)
*#> # A data frame: 3 × 2*
*#> package sysreqs*
*#> <chr> <chr>*
*#> 1 s2 OpenSSL >= 1.0.1*
*#> 2 sf GDAL (>= 2.0.1), GEOS (>= 3.4.0), PROJ (>= 4.8.0), sqlite3*
*#> 3 units udunits-2*

However, sf is a necessary for the full functions of ggVennDiagram in shape generation. So it is difficult to remove it. Therefore, we decided to move the shape generation functions to a new package, namely shapeMageR. And only import this package as a “suggestion” in new version of ggVennDiagram.

Besides, several other packages are removed from dependency list after considerations.

**Current dependency of ggVennDiagram**

deps_new = pak**::pkg_deps**(".")
dependency_new = **c**(
 "ggplot2",
 "dplyr",
 "methods",
 "tibble",
 "aplot",
 "forcats",
 "venn",
 "yulab.utils")
primary_deps = deps_new **|>**
 **select**(package, sysreqs, filesize) **|>**
 **arrange**(**desc**(filesize)) **|>**
 **filter**(package **%in%** dependency_new)
primary_deps
*#> # A data frame: 7 × 3*
*#> package sysreqs filesize*
*#> <chr> <chr> <int>*
*#> 1 ggplot2 "" 4309115*
*#> 2 dplyr "" 1595883*
*#> 3 tibble "" 684102*
*#> 4 forcats "" 423085*
*#> 5 venn "" 309176*
*#> 6 yulab.utils "" 77197*
*#> 7 aplot "" 64276*

**History of dependencies of ggVennDiagram**

Firstly, write a function to do this task.

*#' Report package dependency*
*#'*
*#' @param pkg*
*#' @param size_cutoff*
*#' @param n*
*#' @param exclude*
*#'*
*#' @return*
*#' @export*
*#'*
*#' @examples*
*#' report_package_dependency("ggplot2")*
report_package_dependency = **function**(pkg, size_cutoff = 2**^**20, n = 3, exclude = NULL){
 *# find deps of excluding package*
 **if** (**!is.null**(exclude)){
 **if** (**is.vector**(exclude) **&** **length**(exclude) **>** 1){
 exclude = **lapply**(exclude, **function**(x){
 pak**::pkg_deps**(x) **|>**
 dplyr**::pull**(package)
 }) **|>**
 **unlist**() **|>**
 **unique**()
 } **else** {
 exclude = pak**::pkg_deps**(exclude) **|>** **pull**(package)
 }
 }

 *# find deps and format output*
 deps = pak**::pkg_deps**(pkg) **|>**
 dplyr**::filter**(**!is.na**(ref), **!**(package **%in%** exclude)) **|>**
 dplyr**::arrange**(**desc**(filesize)) **|>**
 dplyr**::rowwise**() **|>**
 dplyr**::mutate**(package_size = **paste0**(package, " (", scales**::number_bytes**(filesize, units = "si"), ")"))

 *# generating report*
 tot_filesize = **sum**(deps**$**filesize, na.rm = TRUE) **|>** scales**::number_bytes**(units = "si")
 big_pkg = deps **|>** dplyr**::filter**(filesize **>** size_cutoff)
 sys_pkg = deps **|>** dplyr**::filter**(sysreqs **!=** "", **!is.na**(sysreqs),
 **!**package **%in%** big_pkg**$**package)
 glue**::glue**("Summary of '{pkg}' Dependency",
 "",
 " Total size: {tot_filesize}; {nrow(deps)} package(s), and {nrow(sys_pkg)} system requirement(s).",
 " Package(s) larger than {scales::number_bytes(size_cutoff, units = 'si')} ({nrow(big_pkg)}): {paste(head(big_pkg$package_size,n), collapse = ', ')}{ifelse(nrow(big_pkg)>n, '...', '.')}",
 " Additional system dependency ({nrow(sys_pkg)}): {paste(head(sys_pkg$package,n), collapse = ', ')}{ifelse(nrow(sys_pkg)>n, '...', '.')}",
 .sep = "**\n**")
}

**for** (tag **in** **c**("V0.5.0","V1.0.7","V1.1","V1.2","V1.2.2","V1.4.9")){
 **report_package_dependency**(**paste0**("gaospecial/ggVennDiagram@", tag)) **|>** **print**()
}
*#> Summary of 'gaospecial/ggVennDiagram@V0.5.0' Dependency*
*#>*
*#> Total size: 160 MB; 50 package(s), and 1 system requirement(s).*
*#> Package(s) larger than 1 MB (20): sf (87 MB), stringi (15 MB), s2 (12 MB)...*
*#> Additional system dependency (1): units.*
*#> Summary of 'gaospecial/ggVennDiagram@V1.0.7' Dependency*
*#>*
*#> Total size: 210 MB; 90 package(s), and 4 system requirement(s).*
*#> Package(s) larger than 1 MB (34): sf (87 MB), stringi (15 MB), s2 (12 MB)...*
*#> Additional system dependency (4): units, tweenr, curl...*
*#> Summary of 'gaospecial/ggVennDiagram@V1.1' Dependency*
*#>*
*#> Total size: 211 MB; 92 package(s), and 4 system requirement(s).*
*#> Package(s) larger than 1 MB (34): sf (87 MB), stringi (15 MB), s2 (12 MB)...*
*#> Additional system dependency (4): units, tweenr, curl...*
*#> Summary of 'gaospecial/ggVennDiagram@V1.2' Dependency*
*#>*
*#> Total size: 210 MB; 90 package(s), and 4 system requirement(s).*
*#> Package(s) larger than 1 MB (34): sf (87 MB), stringi (15 MB), s2 (12 MB)...*
*#> Additional system dependency (4): units, tweenr, curl...*
*#> Summary of 'gaospecial/ggVennDiagram@V1.2.2' Dependency*
*#>*
*#> Total size: 210 MB; 91 package(s), and 4 system requirement(s).*
*#> Package(s) larger than 1 MB (34): sf (87 MB), stringi (15 MB), s2 (12 MB)...*
*#> Additional system dependency (4): units, tweenr, curl...*
*#> Summary of 'gaospecial/ggVennDiagram@V1.4.9' Dependency*
*#>*
*#> Total size: 60 MB; 48 package(s), and 1 system requirement(s).*
*#> Package(s) larger than 1 MB (17): stringi (15 MB), Matrix (5 MB), ggplot2 (4 MB)...*
*#> Additional system dependency (1): fs.*

**report_package_dependency**(".")
*#> Summary of '.' Dependency*
*#>*
*#> Total size: 43 MB; 46 package(s), and 1 system requirement(s).*
*#> Package(s) larger than 1 MB (15): Matrix (5 MB), ggplot2 (4 MB), mgcv (4 MB)...*
*#> Additional system dependency (1): fs.*

**report_package_dependency**(".", exclude = **c**("ggplot2","dplyr"))
*#> Summary of '.' Dependency*
*#>*
*#> Total size: 6 MB; 15 package(s), and 1 system requirement(s).*
*#> Package(s) larger than 1 MB (1): patchwork (3 MB).*
*#> Additional system dependency (1): fs.*

# Data S2 Example for Venn Calculator

Methods for calculating subset members.

**set.seed**(20231225)
y = **list**(
 A = **sample**(letters, 8) **|>** **sort**(),
 B = **sample**(letters, 8) **|>** **sort**(),
 C = **sample**(letters, 8) **|>** **sort**(),
 D = **sample**(letters, 8) **|>** **sort**())

*# view the list*
y
*#> $A*
*#> [1] "a" "e" "g" "o" "p" "s" "t" "v"*
*#>*
*#> $B*
*#> [1] "a" "d" "f" "i" "k" "s" "y" "z"*
*#>*
*#> $C*
*#> [1] "b" "g" "k" "o" "r" "s" "u" "w"*
*#>*
*#> $D*
*#> [1] "b" "c" "e" "h" "k" "q" "s" "y"*

venn_y = **Venn**(y)

venn_y
*#> An object of class 'Venn':*
*#> Slots: sets, names;*
*#> No. Sets: 4 SetNames: A, B, C, D.*

*# find the overlaping members of two or more sets*
**overlap**(venn_y, 1**:**2) *# members in both the first two sets*
*#> [1] "a" "s"*
**overlap**(venn_y) *# members in all the sets*
*#> [1] "s"*

*# find the different members between sets and set unions*
**discern**(venn_y, 1) *# members in set 1, but not in the resting sets*
*#> [1] "p" "t" "v"*
**discern**(venn_y, **c**("A","B"), 3) *# members in set A & B, but not in the 3rd set*
*#> [1] "a" "e" "p" "t" "v" "d" "f" "i" "y" "z"*

*# find the specific members in one or more sets*
**discern_overlap**(venn_y, 1) *# specific items in set 1*
*#> [1] "p" "t" "v"*
**discern_overlap**(venn_y, 1**:**2) *# specific items in set 1 and set 2*
*#> [1] "a"*
